# Age-related changes in brain activity are specific for high order cognitive processes during successful encoding of information in working memory

**DOI:** 10.3389/fnagi.2015.00075

**Published:** 2015-05-11

**Authors:** Diego Pinal, Montserrat Zurrón, Fernando Díaz

**Affiliations:** Applied Cognitive Neuroscience Group GI-1807-USC, Facultade de Psicoloxia, Universidade de Santiago de CompostelaSantiago de Compostela, Spain

**Keywords:** event-related potentials, working memory, encoding, aging, executive functions, slowing of processing

## Abstract

Memory capacity suffers an age-related decline, which is supposed to be due to a generalized slowing of processing speed and to a reduced availability of processing resources. Information encoding in memory has been demonstrated to be very sensitive to age-related changes, especially when carried out through self-initiated strategies or under high cognitive demands. However, most event-related potentials (ERP) research on age-related changes in working memory (WM) has used tasks that preclude distinction between age-related changes in encoding and retrieval processes. Here, we used ERP recording and a delayed match to sample (DMS) task with two levels of memory load to assess age-related changes in electrical brain activity in young and old adults during successful information encoding in WM. Age-related decline was reflected in lower accuracy rates and longer reaction times in the DMS task. Beside, only old adults presented lower accuracy rates under high than low memory load conditions. However, effects of memory load on brain activity were independent of age and may indicate an increased need of processing after stimulus classification as reflected in larger mean voltages in high than low load conditions between 550 and 1000 ms post-stimulus for young and old adults. Regarding age-related effects on brain activity, results also revealed smaller P2 and P300 amplitudes that may signal the existence of an age dependent reduction in the processing resources available for stimulus evaluation and categorization. Additionally, P2 and N2 latencies were longer in old than in young participants. Furthermore, longer N2 latencies were related to greater accuracy rates on the DMS task, especially in old adults. These results suggest that age-related slowing of processing speed may be specific for target stimulus analysis and evaluation processes. Thus, old adults seem to improve their performance the longer they take to evaluate the stimulus they encode in visual WM.

## Introduction

There is a working memory (WM) decline as we age (for review, see Park et al., 2002; Glisky, 2007; Fabiani, 2012). This capacity has been defined as the ability to hold in mind and/or manipulate for brief periods of time small amounts of information that are no longer available in our environment (Baddeley and Hitch, 1974; Baddeley, 2012). Additionally, it is thought to comprise three different cognitive events: information encoding, maintenance, and retrieval (for a review see Jonides et al., 2008).

Working memory decline due to normal aging processes is indicated by lower accuracy rates and longer reaction times (RTs) in performance of experimental tasks (Baltes et al., 1999; Park et al., 2002). Several explanatory hypotheses have been proposed to account for this decline. For instance, it has been suggested that it may be caused by a general slowing of processing speed in old adults (Salthouse, 1996; Rousselet et al., 2009), and/or by deficits in frontal lobe function, which, in turn, give rise to alterations in executive control (Hasher and Zacks, 1988; West, 1996; Paxton et al., 2008; Kalkstein et al., 2011).

Recent evidence have led several authors to suggest that such age-related decline in WM is mainly caused by changes in brain activity during information encoding (Friedman et al., 2007; Finnigan et al., 2011; Craik and Rose, 2012), especially when the encoding strategies are self-initiated, i.e., without specific instructions about how to encode information (Hashtroudi et al., 1989; Friedman et al., 2007; Craik and Rose, 2012). Moreover, differences in mnemonic capacities associated with normal aging are enhanced in tasks that impose high demands in cognitive abilities, such as those in which memory load is manipulated (Oberauer and Kliegl, 2001; Buckner, 2004; Gazzaley et al., 2007). Indeed, some authors have suggested that old adults are more sensitive to cognitive demands than young adults, and they therefore show signs of cognitive effort at lower levels of demand than young adults (Park and Reuter-Lorenz, 2009; Cabeza and Dennis, 2013).

The use of event-related potentials (ERP) enables analysis of electrical brain activity with a temporal resolution and precision in the order of a few milliseconds. Consequently, this technique is a potentially useful tool for studying the different neural processes underlying information encoding in WM. However, most ERP research focusing on age-related changes in WM employed *n*-back tasks, which, unfortunately, preclude distinction between information encoding and information retrieval. Nevertheless, this can be overcome by using other tasks such as Sternberg’s (1966) tasks and delayed match to sample tasks (DMS; John et al., 1996), both of which include a stage when information must be encoded and actively held in mind and a separate later stage when information must be retrieved in order to complete some kind of judgment (e.g., same vs. different, present vs. absent, …).

Using the Sternberg (1966) paradigm, Finnigan et al. (2011) found that for words that have to be encoded in WM, the P1 latency was longer and N1 amplitude was larger in old adults than in young adults. However, during the information encoding stage in a DMS task, Gazzaley et al. (2008) and Zanto et al. (2010) did not observe any age-related differences in P1 component latency or amplitude, although they observed longer N1 latencies. Also in contrast to the findings of Zanto et al. (2010) and Finnigan et al. (2011), reported lower N1 amplitude in old than young adults. Consequently, despite longer P1 (Finnigan et al., 2011) or N1 (Gazzaley et al., 2008; Zanto et al., 2010) latencies for old than for young participants have been interpreted as evidence for a generalized slowing of processing speed in old adults, age-related effects on these ERP components during information encoding in WM are still unclear due to the partly contradictory results of previous studies.

Furthermore, to our knowledge, no studies have assessed the possible interactions between memory load and the age-related effects on these ERP components, which have been shown to be sensitive to memory load in young adults during WM encoding (Morgan et al., 2008).

Regarding ERP components that have been related to high order cognitive processes, several studies using *n*-back tasks have demonstrated that fronto-central P2 and N2 components are sensitive to aging effects on WM processes (McEvoy et al., 2001; Missonnier et al., 2004, 2011). However, there is some controversy. That is, while McEvoy et al. (2001) observed higher P2 amplitude in old adults, Missonnier et al. (2004, 2011) found that the area delimited jointly by P2 and N2 components was lower in old than in young adults. Furthermore, in McEvoy et al. (2001) study, memory load had no effects on P2 amplitude, whereas the area under P2-N2 complex increased with memory load in young but not in old adults in Missonnier et al. (2004, 2011) experiments. Hence, the effect of any interaction between memory load and aging on these components amplitude is also unclear.

Concerning the P300 component, in studies involving *n*-back tasks, McEvoy et al. (2001) and Saliasi et al. (2013) observed longer latencies and lower amplitudes, at parietal electrodes, for old than young adults. In a DMS task, Gazzaley et al. (2008) observed longer P300 latency in old than young participants. These results are consistent with the effects of age on P300 ERP component that has been repeatedly validated regardless of the task used (for reviews see Polich, 1996; Friedman, 2012). Such age-related effects on P300 have been considered to indicate a reduction in the availability of processing resources and of a slowing in stimulus evaluation and categorization (McEvoy et al., 2001; Lorenzo-López et al., 2008; Saliasi et al., 2013). Similarly, decreased P300 amplitude and increased latency have been consistently found with increasing memory load (for review see Kok, 2001; Polich, 2007). However, most studies reporting such results only involved young adult samples.

Although the above findings are promising with respect to the use of ERPs as a tool for assessing age-related effects on the neural processing underlying information encoding in WM, they are not conclusive. Therefore, the aim of the present study was to answer some questions in relation to the effects of aging on the time course of electrical brain activity associated with the self-initiated successful encoding of visual information in WM. The specific aims were to determine: (i) whether there is an age-related slowing in the behavioral response to a memory task and in the ERP indexes of the cognitive processes involved in visual information encoding in WM; (ii) which stages of information encoding processing suffer an age-related reduction in allocation of processing resources; and (iii) the effect of memory load and its interaction with age-related effects on task execution and in ERP components related to information encoding in WM.

For these purposes, ERPs were measured in a sample of healthy young and old adults while they performed a DMS task involving presentation of a stimulus that had to be memorized (encoding stage) through self-initiated strategies. Moreover, the stimuli presented correspond to two different levels of memory load.

## Materials and Methods

### Sample

The sample comprised 40 volunteers. All except three were right handed, as assessed by the Edinburgh Handedness Inventory (Oldfield, 1971). All participants had normal or corrected-to-normal vision and reported no history of neurological or psychiatric disorders. In addition, all participants, none of whom were taking psychotropic medication, were instructed to abstain from consuming alcohol and caffeine the day before the experimental session. All volunteers gave their informed consent to participating in the study, and the study protocol was approved by the ethical Committee at the University of Santiago de Compostela (USC).

Participants were further divided in two groups (each with 16 females): 20 young adults (mean age = 23.85 ± 3.18 years) recruited from USC alumni, and 20 healthy old adults (mean age = 67.80 ± 7.69 years) recruited from USC courses for older adults and from two different cultural associations in which they participate in cognitively demanding activities (learning a foreign language, informatics courses, etc.). The two groups significantly differ in time spent in formal education (young: 16.25 ± 1.25 years, old: 14.12 ± 3.93 years; *t*(18.79) = -2.160, *p* ≤ 0.044) but were equated in their scores on the Spanish version of the Wechsler Adults Intelligence Scale vocabulary subtest (Wechsler, 1997; young: 47.95 ± 5.22, old: 48.15 ± 8.68; *t*(31.41) = 0.89, *p* ≤ 0.930).

### Experimental Protocol

**FIGURE 1:**
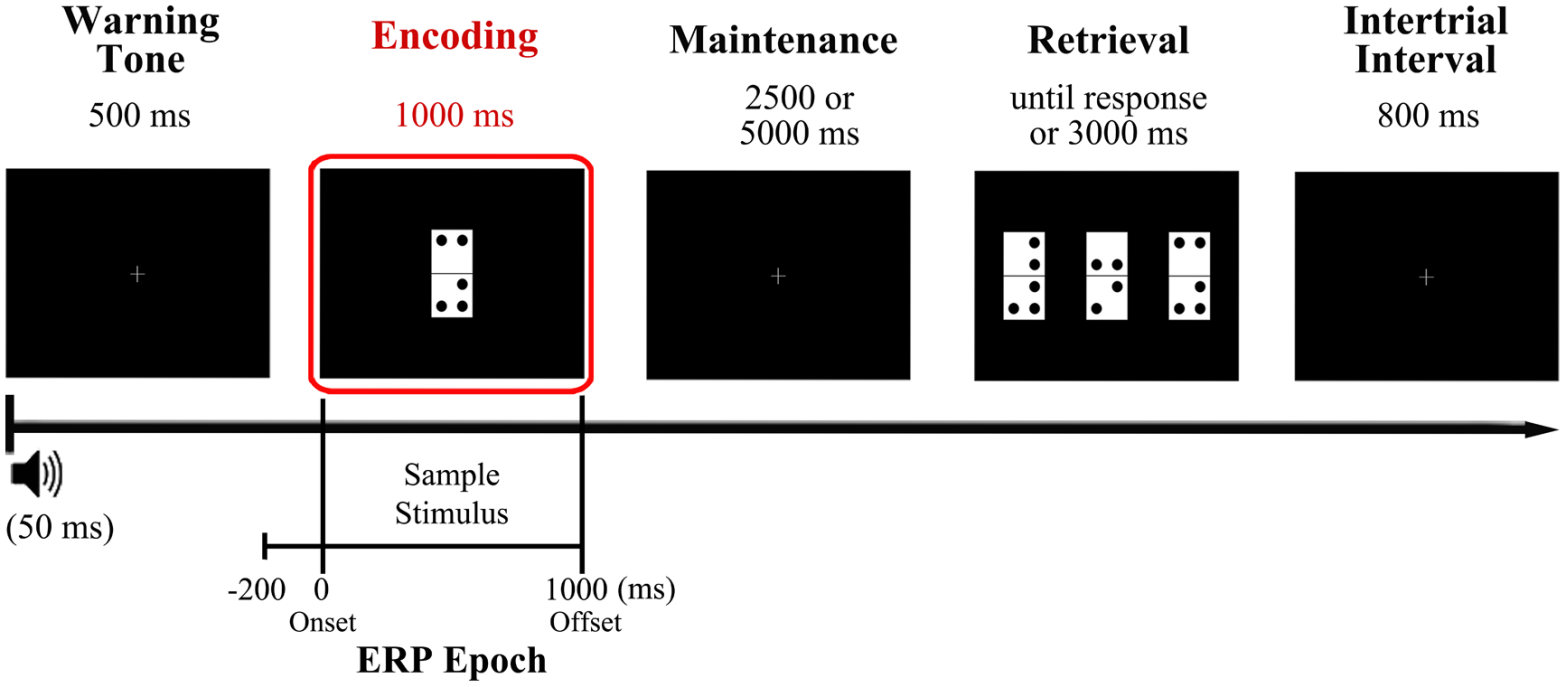
**Diagram of the delayed match to sample (DMS) task used in the study and time reference for the analyzed event-related potentials (ERP) epoch**. Participants were presented with a domino tile, the configuration of which they had to hold in mind for a variable delay (2.5 or 5 s) before identifying it as quickly and accurately as possible from among three different options presented simultaneously at the retrieval stage. The encoding phase, which was the focus of the present study, is highlighted in red. The analyzed ERP epoch extends from 200 ms prior to sample stimulus onset (during warning tone period) to 1000 ms after, coinciding with sample stimulus offset.

Participants performed the visual DMS task illustrated in **Figure 1**, which is described in detail elsewhere (Pinal et al., 2014). They were asked to memorize a domino tile presented as sample stimulus, retain its identity for a brief delay of several seconds, and identify as quickly and accurately as possible the memorized domino among three different domino tiles presented as probe stimulus, with only one of them being identical to sample stimulus (target).

More in detail, a warning tone (1000 Hz pitch, 50 ms duration) was used to indicate the start of each trial and was followed, 500 ms later, by presentation of a sample stimulus, which remained on the screen for 1000 ms. This was followed by a blank screen delay of 2500 or 5000 ms (50% of probability of appearance) and then by the presentation of three new dominoes as probe stimuli. The tiles remained on screen until the participants responded or for a maximum time of 3000 ms. The response was performed pressing the button corresponding to the position of the target on screen (left, center, or right: this was counterbalanced across trials so it never appeared more than three consecutive trials in the same position) out of three response buttons arranged horizontally on a response device (Cedrus^®^, model RB-530). The inter-trial interval duration was 800 ms. To minimize ocular artifacts, a fixation cross was placed in the center of the screen when no stimuli were presented. Stimuli presentation and response recording were controlled using Presentation^®^ software (Neurobehavioral Systems, Inc., Albany, CA, USA). After receiving a brief training in the task, participants completed a total of 200 trials divided in two blocks separated by a 5 min interval.

The domino tiles (length, 8 cm and width, 4 cm) comprised two vertically arranged white squares of equal size. They were marked with between two and five black dots (1 cm in diameter) at the corners of each square, leaving with a gap of 1 cm between each dot and a gap of 0.5 cm between each dot and the edges of the squares. The domino tiles were presented on a black background in the center of a monitor (19′′, refresh rate of 100 Hz) located at a distance of 1 m from the participant’s eyes, so that each domino subtended a visual angle of 4.58° × 2.28°.

Memory load was manipulated between trials by changing the number of dots on the dominoes, which were grouped into two memory load conditions: low load condition (LL) corresponding to dominoes with two or three dots, and a high load condition (HL) corresponding to dominoes with four or five dots. Dominoes were grouped in the aforementioned two memory load conditions to ensure an adequate and homogenous number of epochs for the ERP analyses. Besides, the three dominoes that comprise the probe stimulus all belong to the same memory load condition as the sample stimulus. The first block of trials consisted of 90 low memory load trials, while the second block consisted of 110 high memory load trials. The percentage of repeated dominoes was maintained constant in both blocks (20%). The HL block included more trials than the LL block to ensure a good signal-to-noise ratio, since a higher proportion of errors was expected for this block.

Note that, for this study, only the successful encoding of the sample stimulus into WM was of interest. Therefore, only parameters of the ERP related to the presentation and processing of the sample stimulus (that has to be encoded in WM) in correctly responded trials were analyzed.

### EEG Recordings and Signal Processing

During the experimental session, participants sat in a comfortable armchair inside a noise and light attenuated Faraday chamber. EEG activity was recorded at 51 active electrodes inserted in a cap and placed in the standard positions of the 10–10 system. Fronto-polar ground and nose tip reference were used, with all the electrode impedances maintained below 10 kΩ. EOG activity was also monitored with two electrodes placed at the outer canthi of both eyes (HEOG) and another two electrodes placed above and below the right eye (VEOG). The EEG signal was analogically filtered between 0.01 to 100 Hz, sampled at 500 Hz and digitally recorded for off-line analysis.

Recorded data were passed through a digital phase-shift free Butterworth filter with the high cut-off frequency at half power (-3 dB) set at 30 Hz (12 dB/octave roll-off), and with a low cut-off frequency at half power set at 0.1 Hz (12 dB/octave roll-off). A notch-filter centered at 50 Hz was also applied to avoid any contamination of electrical line noise. Ocular and muscular artifacts were corrected using the Infomax algorithm in an Independent Component Analysis (ICA) as implemented in Brain Vision Analyzer (v.2 Brain Products GmbH). Furthermore, a semi-automatic artifact rejection was conducted (i.e., trials with voltage changes of ±125 mV were excluded). Data was then segmented in epochs from 200 ms prior to sample stimulus presentation to 1000 ms post-stimulus, and baseline was corrected with the mean activity in the 200 ms prior to sample stimulus (**Figure 1**). Only epochs corresponding to correctly answered trials entered further analyses.

### Behavioral and Electrophysiological Data

The proportion of correct responses and RTs for the correctly responded trials were recorded for each participant and experimental condition (LL and HL).

As regards electrophysiological data, separate averaged ERP waveforms to sample stimulus were obtained for each memory load condition. The following components were identified: P1, N1, P2, N2, and P300. On the basis of the reports reviewed in the introduction section and by choosing the electrodes where amplitude was maximal, peak latency, and baseline to peak amplitude of those components were measured as follows: the P1 component was considered the maximum peak at O1, Oz, and O2 between 85 and 145 ms post-stimulus; N1 was defined as the most negative peak at P9, P7, P8, P10, between 150 and 210 ms post-stimulus; the P2 component was measured as the largest positive peak at F3, Fz, and F4 between 180 and 250 ms post-stimulus; N2 was considered the maximum negative-going peak in the trough between P2 and P300 positive waves at F3, Fz, F4, C3, Cz, and C4 between 230 and 300 ms after sample stimulus onset; and P300 was identified as the maximum positive peak at P3, Pz, and P4 between 300 and 500 ms post-stimulus. For each component latency and amplitude data were averaged across the selected electrodes. The statistical tests were applied to mean values for each component.

**FIGURE 2:**
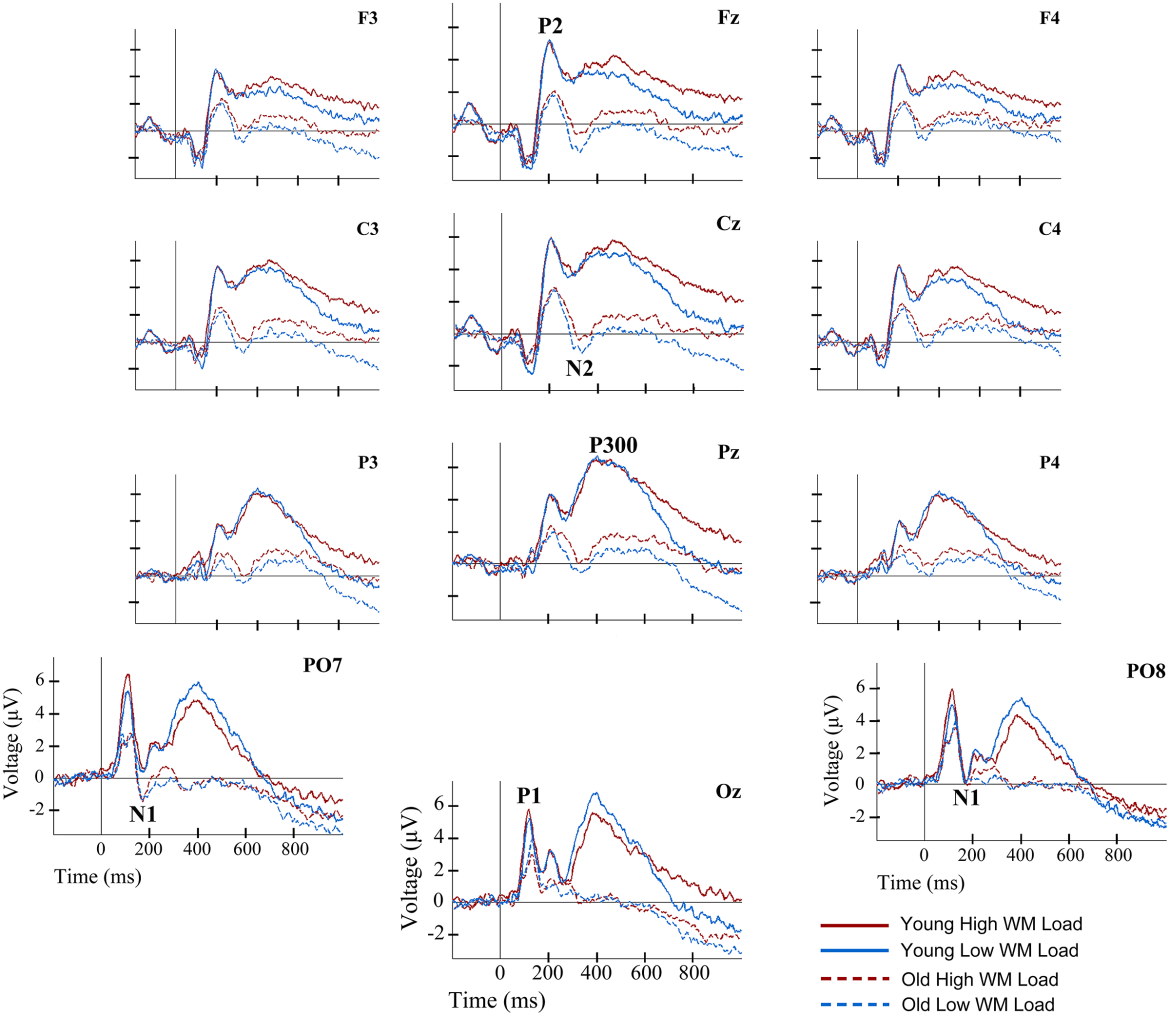
**Event-related potentials waveforms for young and old groups, and for two load conditions, at several electrode locations during information encoding in working memory (WM)**. Solid lines represent the ERP waveforms of young adults during encoding of information in WM. Dashed lines depict the ERP waveforms of old adults during information encoding in WM. Red lines correspond to the high load (HL) condition. Blue lines correspond to the low load (LL) condition.

In addition, because the N2 component did not reach negative voltage values in the young participants, in contrast to the older participants (see **Figure 2**), it was considered appropriate to measure N2 amplitude with respect to the previous and posterior positive peaks. In other words, in addition to the baseline to peak amplitude, the N2 amplitude was measured from peak to trough (P2-N2) and from trough to peak (N2-P300). These amplitude measures were made at the same electrodes as described earlier for N2. The mean amplitude across the selected electrodes was used in the statistical analysis.

Finally, as no ERP component peak was clearly identifiable from 500 ms after stimulus onset onward, the mean amplitudes for three different time windows were obtained for frontal and parietal midline electrodes. The first time window spanned from 550 to 700 ms, the second one extended from 701 to 850 ms post-stimulus, and the final time window spanned from 851 to 1000 ms after stimulus onset.

### Statistical Analyses

Mixed design analyses of variance (ANOVA) with Age Group (young and old) as the between subjects factor and Memory Load (LL and HL) as the within-subject factor were carried out with behavioral performance data (i.e., RTs and accuracy rates), as well as with the parameters (peak latency and amplitude values) of the ERP components, and P2–N2 and N2–P300 amplitudes. Region (frontal and parietal) as within subjects factor was also included for the ANOVAs carried out with the mean amplitude values in the time intervals 550–700, 701–850, and 851–1000 ms post-stimulus.

For all ANOVAs performed in the present work, Greenhouse–Geisser correction was applied whenever the sphericity assumption was violated, and Bonferroni adjustment was used to correct for multiple comparisons.

Finally, to assess the relationship between electrical brain activity and behavioral performance independently of age (specifically, to determine which neural processes at encoding were related to task execution), partial correlation analysis controlling for age (Finnigan et al., 2011; Amenedo et al., 2012) was executed between behavioral outputs (RTs and accuracy rates) and latency or amplitude values of the ERP components. Behavioral outputs and ERP parameters were averaged across load conditions prior to being entered in the partial correlation analyses. Bootstrapping (1000 samples) was used to control for multiple comparisons.

Pearson’s correlations were also calculated separately for each age group between the behavioral outputs and the ERP components parameters that were significantly correlated when age was partialled out.

In all statistical tests, differences were considered statistically significant at *p* ≤ 0.05.

## Results

### Behavioral Performance

The ANOVAs of task performance data (i.e., accuracy rates and RTs) revealed a significant effect for Age Group factor in both measures ([*F*(1,38) = 24.73, *p* < 0.001] and [*F*(1,38) = 59.04, *p* < 0.001], respectively). Therefore, old adults showed significantly lower accuracy rates and significantly longer RT relative to young adults (**Table 1**).

**Table 1:** Behavioral data.

	Old		Young	
	Low load (LL)	High load (HL)	Low load (LL)	High load (HL)
Reaction time (RT)	1530.1 ± 208.8	1668.2 ± 175.6	1079.8 ± 208.7	1187.8 ± 220.3
Accuracy	89.9 ± 8.9	78.8 ± 13.1	96.9 ± 3.2	93.7 ± 3.7

*RT (in milliseconds) and percentage accuracy for each group and memory load condition. Mean value ± Standard Deviation.*

In addition, the interaction between the factors Age Group and Memory Load also had a statistically significant effect on accuracy rates [*F*(1,38) = 16.32, *p* < 0.001], with significantly lower accuracy rates in the HL than the LL condition only in the old adults group (*p* < 0.001).

### Electrical Brain Activity

For P1 and N1 amplitude and latency, the ANOVAs did not reveal any significant effect of Age Group, Memory Load, or of their interaction.

**FIGURE 3:**
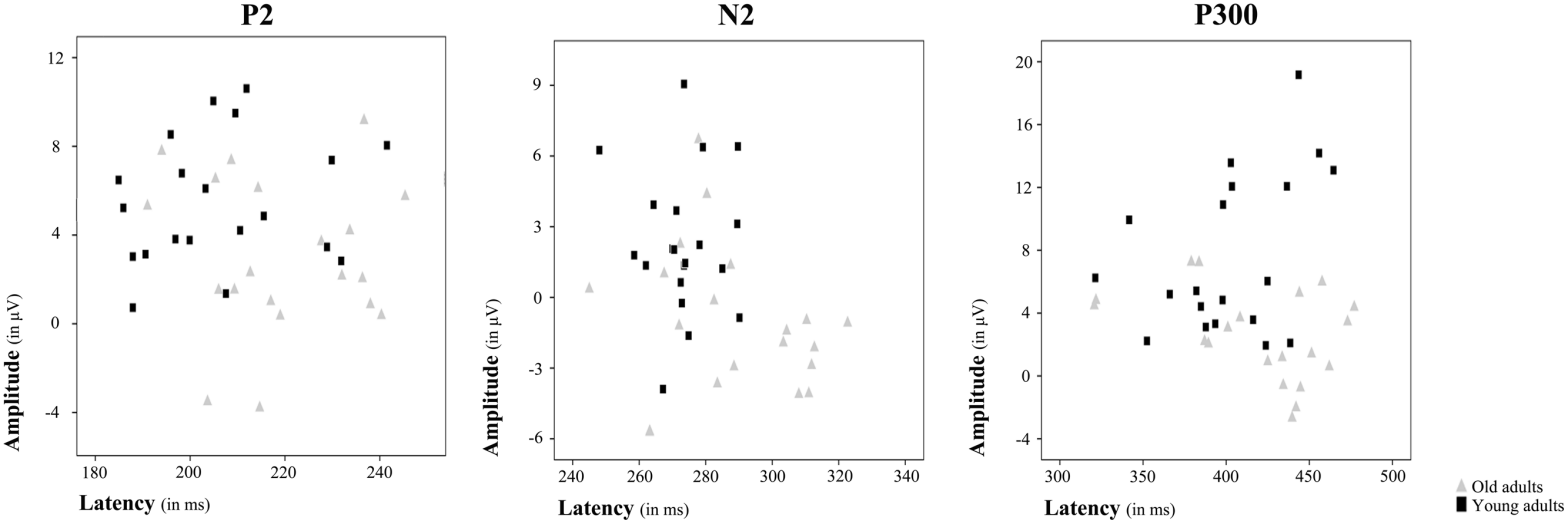
**Amplitude and latency plots for each ERP component showing differences between age groups**. Scatterplots present individual amplitude and latency values for P2, N2, and P300, respectively. Each gray triangle represents amplitude and latency for an individual old adult and each black square represents amplitude and latency for an individual young adult.

Regarding the P2 component, a main effect of Age Group was found for its latency [*F*(1,38) = 6.36, *p* ≤ 0.016] and amplitude [*F*(1,38) = 7.79, *p* ≤ 0.008]. Thus, the P2 latency was significantly longer in the old adults than in the young adults, while amplitude was significantly lower in old adults than in the young adults (**Figures 2** and **3**).

The Age Group factor also had a significant effect on N2 latency [*F*(1,38) = 8.19, *p* ≤ 0.007] and amplitude [*F*(1,38) = 11.71, *p* ≤ 0.002]. The latency of N2 was significantly longer and the N2 amplitude was significantly larger in the older adults than in the young participants (**Figures 2** and **3**). However, P2-N2 and N2-P300 peak to peak amplitudes were not significantly affected by the factor Age Group. No significant effects were observed for Memory Load factor or the interaction between Memory Load and Age Group on N2 peak latency or amplitude, or on P2-N2 and N2-P300 peak to peak amplitudes.

Regarding the P300 component, latency values did not differ significantly between the two age groups, although as can be seen in **Table 2**, old adults presented longer latencies than young adults. Regarding the P300 amplitude, there was a main effect of Age Group [*F*(1,38) = 15.43, *p* < 0.001], with significantly lower amplitude in the old adults than in the young adults (**Figures 2** and **3**). Neither Memory Load factor nor its interaction with Age Group had statistically significant effects on P300 latency and amplitude.

**Table 2:** Parameters of ERP components.

		Old		Young	
		Low load	High load	Low load	High load
P1	*Latency*	121.7 ± 3.2	120.8 ± 3.8	117.7 ± 2.3	115.6 ± 2.8
	*Amplitude*	5.3 ± 0.9	4.6 ± 0.8	6.3 ± 0.9	7.0 ± 1.0
N1	*Latency*	175.9 ± 3.4	174.7 ± 3.3	167.2 ± 3.5	170.4 ± 3.2
	*Amplitude*	-1.7 ± 0.5	-1.6 ± 0.6	-1.4 ± 0.7	-1.1 ± 0.6
P2	*Latency*	220.6 ± 3.9	218.0 ± 4.0	208.5 ± 4.5	204.1 ± 3.4
	*Amplitude*	2.9 ± 0.8	3.2 ± 0.8	5.9 ± 0.6	5.6 ± 0.6
N2	*Latency*	287.6 ± 4.4	289.1 ± 5.9	270.7 ± 2.9	275.7 ± 3.5
	*Amplitude*	-1.4 ± 0.8	-0.7 ± 0.7	2.2 ± 0.7	2.4 ± 0.7
P3	*Latency*	422.8 ± 10.5	414.6 ± 12.4	401.0 ± 10.7	402.9 ± 9.0
	*Amplitude*	2.2 ± 0.8	3.0 ± 0.6	7.8 ± 1.1	7.5 ± 1.2
550 –700 ms	*F*z *Amplitude*	-0.5 ± 0.6	0.3 ± 0.6	2.5 ± 0.9	3.2 ± 0.9
	*P*z *Amplitude*	0.2 ± 0.6	1.4 ± 0.6	3.4 ± 1.1	3.4 ± 1.2
701 – 850 ms	*F*z *Amplitude*	-0.8 ± 0.8	0.1 ± 0.7	1.0 ± 0.8	2.3 ± 0.8
	*P*z *Amplitude*	-0.7 ± 0.8	0.7 ± 0.7	0.6 ± 1.0	2.4 ± 1.3
851–1000 ms	*F*z *Amplitude*	-1.2 ± 0.9	-0.0 ± 0.8	0.7 ± 0.8	1.9 ± 0.8
	*P*z *Amplitude*	-1.9 ± 0.9	-0.2 ± 0.7	-0.3 ± 0.9	1.4 ± 1.2

*Latency (in milliseconds) and amplitude (in μV) for each group and memory load condition. Mean value ± Standard Error.*

Finally, the ANOVAs revealed that Age Group had a main effect on mean amplitude in the 550–700 ms interval [*F*(1,38) = 7.21, *p* ≤ 0.011], when amplitude was significantly lower in old than young adults. In the 701–850 ms and 851–1000 ms time intervals, a main effect of Memory Load was found [*F*(1,38) = 13.01, *p* ≤ 0.001 and *F*(1,38) = 14.37, *p* ≤ 0.001, respectively], with significantly larger mean amplitudes in the HL condition than in the LL trials in both cases (**Figure 2**).

### Relationships between Electrical Brain Activity and Behavioral Performance

**FIGURE 4:**
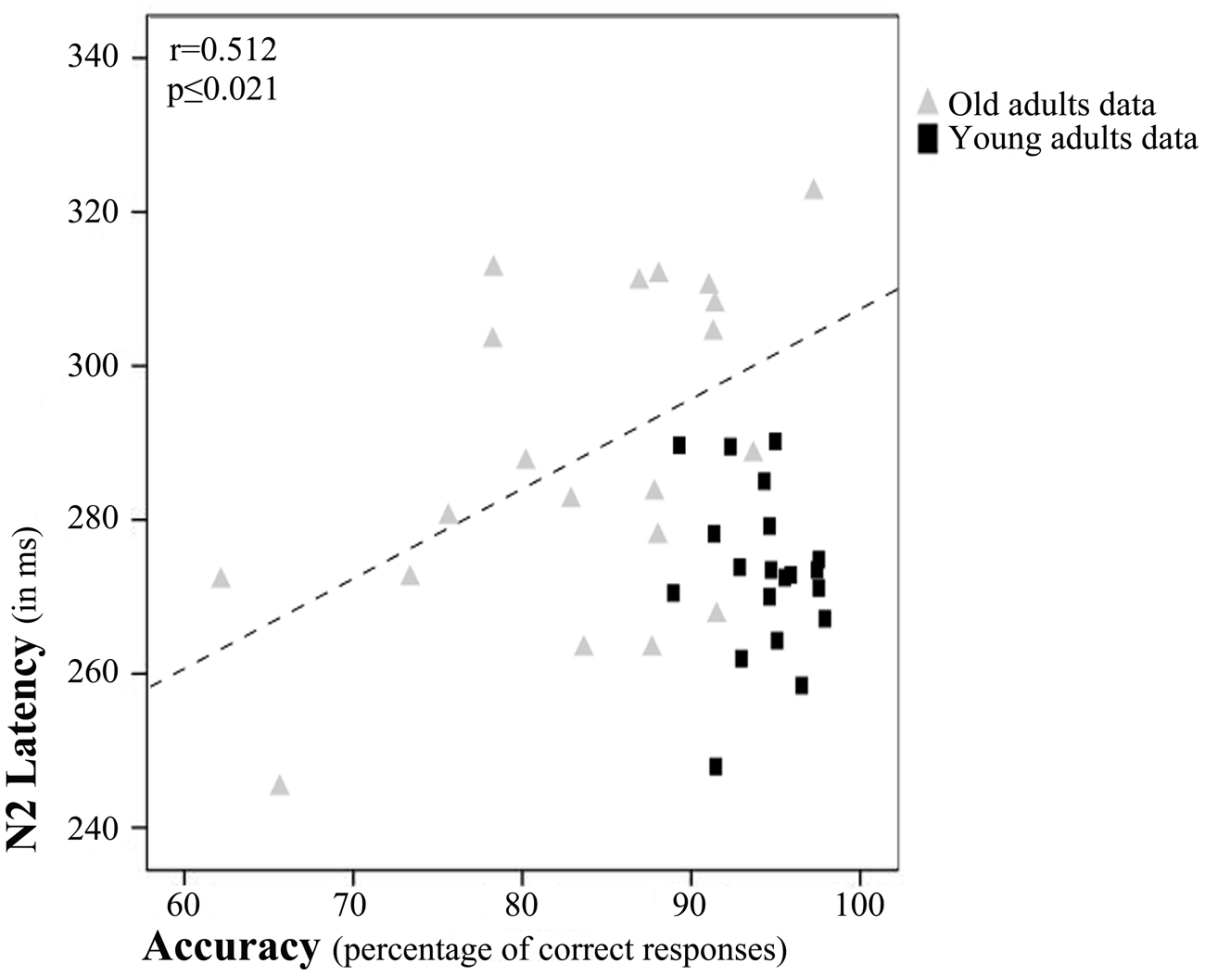
**Relation between N2 latency and accuracy rates**. Scatterplot of data for young and old adults. The depicted best fit line and correlation values (*r* and associated *p*) refer to Pearson’s correlation analysis applied to data for old adults only.

Regarding partial correlation analysis, once the effects of age (chronological) were controlled for, a significant relationship between accuracy rates and N2 latency emerged (*r* = 0.452, *p* ≤ 0.004; **Figure 4**). Thus, the accuracy rates increased with N2 latency.

However, after examination of the scatterplot, we decided to calculate Pearson’s correlations between N2 latency and accuracy rates separately for each age Group. These tests revealed a significant correlation between accuracy rates and N2 latency only in the older adults (*r* = 0.512, *p* ≤ 0.021; **Figure 4**).

## Discussion

### Task Performance

Results revealed age-related differences in performance of the experimental task, as the response of the old participants to the DMS task were less effective (lower accuracy rates) and slower (longer RTs) than those of young adults. In addition, memory load was found to modulate task performance, but only in the old adults, who showed lower accuracy rates when memory load was high than when it was low. The latter result may indicate that old adults are more sensitive than young adults to memory load effects.

### Electrical Brain Activity Related to Information Encoding in WM

#### ERP Latency Measures and Slowing of Processing Speed

Analysis of brain electrical activity during visual information encoding in WM showed that P1, N1, and P300 latencies were not affected by age or memory load. On the other hand, P2 and N2 latencies were longer in old than in young participants, but were not modulated by memory load.

In contrast with the findings of previous studies that used Sternberg (1966) or DMS tasks (Gazzaley et al., 2008; Zanto et al., 2010; Finnigan et al., 2011), there were no differences between old and young adults for P1 and N1 latencies. These components are usually associated with perceptual processing of visual inputs (Kok, 1997; Vogel and Luck, 2000; Taylor, 2002; Itier and Taylor, 2004; Luck, 2012); consequently, differences between the present and previous studies in the type (domino tiles vs. words, faces or a circular aperture of 290 colored dots), number (one domino tile vs. 4–8 words, two faces or two circular apertures of colored dots) and/or on the presentation of stimuli (simultaneous vs. sequential) may explain the discrepancy in the results. Nevertheless, the present results indicate that the age-related slowing of processing speed does not affect perceptual processing of stimuli to be encoded, at least under the experimental conditions used in the present study.

As regards P2 and N2 components, longer latencies were observed for old than for young adults, which is consistent with findings from previous studies that used *n*-back tasks (Missonnier et al., 2004, 2011). Despite the lack of general agreement about its functional role, P2 has been related to a top–down mechanism for rapid evaluation of stimulus significance that facilitates the posterior processing of familiar (James et al., 2001; Paynter et al., 2009) or subjectively relevant stimuli (Schapkin et al., 2000; Potts and Tucker, 2001; Potts, 2004; Gilmore et al., 2009). Moreover, N2 has been considered a correlate of the ease of visual information encoding (Nittono et al., 2007; Ferrari et al., 2010); and its latency has been used as a physiological marker of the timing of access to different properties of a stimulus (Schmitt et al., 2000, 2001a,b; Folstein and van Petten, 2008). Therefore, the present results appear to indicate that in situations like those established in the present study, there is an age-related slowing in stimulus analysis and evaluation during information encoding in WM.

No significant age-related differences were found for P300 latency, which contradicts the results of previous studies (for reviews see Polich, 1996; Friedman, 2012). Nonetheless, P300 latencies were longer in older adults than in young adults, especially in the HL condition (**Table 2**). The fact that in old adults the P300 resembled a plateau with multiple peaks (see **Figure 2**) and that the latency was measured only from the most positive peak may explain the lack of significant differences between both groups.

In summary, the results for ERPs latency showed that the age-related slowing of processing speed is not general during information encoding in WM, since it did not affect initial perceptual processing of the stimulus, but affected processes related to stimulus analysis and evaluation of stimulus features. In addition, during information encoding, memory load did not modulate the latency of the ERP components under study.

#### ERP Amplitude Measures and Allocation of Processing Resources

With respect to the amplitude of the ERP components, no significant effects of age or memory load were found for P1 or N1. However, the analyses revealed lower P2 and P300 amplitudes as well as lower mean amplitude in the 550–700 ms time interval for old than young adults. Moreover, mean amplitudes in 701–850 ms and 851–1000 ms time intervals were significantly larger in high than low memory load conditions in both age groups.

The lack of significant age-related effects on P1 and N1 amplitudes contrasts with previous findings for these components, which have been associated with perceptual analysis of visual inputs (e.g., Gazzaley et al., 2008; Zanto et al., 2010; Finnigan et al., 2011). This is probably due to differences in the type, number, or presentation of stimuli used in this and previous studies. Overall, the results of the present study appear to indicate that under the conditions imposed by the present task, differences in the allocation of processing resources for perceptual processing during visual information encoding in WM are not related to age.

Previous studies concerning aging effects on P2 amplitude showed partly opposite results (McEvoy et al., 2001; Missonnier et al., 2004, 2011). The amplitude of this component, which has itself been repeatedly related to some aspects of stimulus evaluation, has been associated with the amount of processing resources allocated to a rapid top–down evaluation of stimulus significance (Wang et al., 2010). Hence, the lower P2 amplitude found for old than young adults in the present study might indicate an age-related deficit in the allocation of processing resources for the evaluation of stimulus significance.

The N2 peak amplitude was significantly larger in the old than the young group, although P2-N2 and N2-P300 peak to peak amplitudes were not affected by age. Consequently, it appears that age group differences in N2 peak amplitude are driven by the large positive amplitude that P2 and P300 had in the young adults, which, in turn, produce more positive (above baseline) N2 amplitude values for young than old adults.

Previous studies have consistently found an age-related reduction in the amplitude of P300 component at parietal electrodes (McEvoy et al., 2001; Saliasi et al., 2013; for a review see Polich, 1996; Friedman, 2012). This has been interpreted as an age-related decrease in the processing resources available for allocation to stimulus categorization. Likewise, in the present study, the P300 amplitude was lower in old than in young participants, and mean amplitude was also lower in the 550–700 ms time interval, which immediately follows P300 peak. Therefore, the present results provide evidence that, relative to young adults, old adults have lower amounts of processing resources available for the successful categorization of stimuli to be encoded in WM.

Memory load effects were restricted to the mean amplitude for 701–850 ms and 851–1000 ms time intervals, during which mean amplitudes were significantly larger for HL than LL stimuli. These results are consistent with those of García-Larrea and Cézanne-Bert (1998), who interpreted the larger amplitude under HL conditions as an index of extra stimulus processing that is not needed in LL conditions. Furthermore, ERP activity following P300 has been related to active maintenance processes and/or to elaboration of categorized and encoded materials in WM (García-Larrea and Cézanne-Bert, 1998; Chen et al., 2007; Folstein and van Petten, 2011). Thus, it seems that the memory load manipulations in the present study did not interact with age effects on electrical brain activity. However, high memory load may require the allocation of extra processing resources for mental processes once the target stimulus has been categorized and has to be maintained in an active state in WM for its comparison with the probe stimulus.

It is noteworthy that in the present study no memory load effects were found on electrical brain activity before 700 ms. This is at odds with the findings of previous studies in which memory load was manipulated during information encoding in WM (e.g., Morgan et al., 2008; Soria Bauser et al., 2011). The previous studies reported an increase in P1 and decrease in N1 amplitude (Morgan et al., 2008), as well as memory load dependent P300 amplitude reductions during the encoding stage of DMS tasks (Morgan et al., 2008; Soria Bauser et al., 2011). Differences between the present and previous results may depend on how memory load was manipulated (i.e., number of dots inside a single item vs. number of items to be encoded). Also, the fact that the two memory load conditions used in the present study combine domino tiles with a different number of dots (i.e., two and three dotted dominoes in the LL condition and four and five dotted dominoes in the HL condition) may have obscure potential memory load effects as those observed in previous studies. Consequently, in the present study, memory load manipulation (total number of dots on a domino tile) was not sufficient to impose great demands on perceptual processing as well as on stimulus evaluation and categorization cognitive processes. This is supported by the very high accuracy rates for task performance in young adults (close to 95% in both memory load conditions).

In summary, analysis of the amplitude of ERP components revealed that the amount of processing resources allocated to perceptual processing is preserved in old adults; however, there is a decrease in the availability of processing resources for the evaluation and categorization of visual stimuli to be encoded in WM. Moreover, in both age groups high memory load conditions require allocation of processing resources for extra processing after target stimulus categorization has been completed, when maintenance of information in WM and rehearsal processes are carried out.

### Relation between Brain Activity and Task Performance

Partial correlation analysis between the latency or amplitude of ERP components and task performance measures, with age partialled out, showed that brain electrical activity during information encoding in WM is related to DMS task performance. In particular, N2 latency was directly related to accuracy rates (see **Figure 4**). Therefore, higher accuracy rates were associated with longer the N2 latencies. In other words, the longer the time dedicated to feature analysis for the stimuli to be encoded in WM, the better the recognition of those stimuli when presented among two other similar stimuli that acted as distractors.

However, visual inspection of the scatterplot, in which the effect seems to be driven by the data for old adults, prompted us to calculate the Pearson’s correlation between N2 latency and accuracy rates for each group separately. It seems that in the old age group, long evaluation of stimulus features is more beneficial than in young adults. Overall, the results of the correlation analyses might indicate that the longer the time spent in stimulus feature analysis during encoding of information in WM, the better the accuracy rates in a DMS task, especially in old adults.

## Conclusion

The aim of the present study was to evaluate the effect of normal aging on the successful self-initiated encoding of information in WM, as well as to determine the potential interactions between aging and memory load effects. The results showed that high levels of memory load caused a decline in task performance only in old participants. Nevertheless, memory load effects on brain electrical activity were age independent and characterized by an increase in processing resources allocated for information maintenance in WM once the stimulus has been categorized. Regarding normal aging effects, the results revealed an age-related slowing in the response to the memory task and that the age-related slowing of processing speed is not general, since target stimulus analysis and evaluation processes are slower, but perceptual processing is not. The findings also indicated an age dependent decline in the allocation of processing resources for stimulus evaluation and categorization. Finally, correlation analyses showed that brain electrical activity during information encoding in WM is related to task performance. In particular, longer N2 latencies, which are associated with stimulus analysis and evaluation processes, predicted higher accuracy rates in the DMS task, especially in old adults.

## Author Contributions

All three authors contributed to the design and planning of the present study and also cooperated in writing the manuscript. DP was in charge of data acquisition and analysis.

## Conflict of Interest Statement

The authors declare that the research was conducted in the absence of any commercial or financial relationships that could be construed as a potential conflict of interest.
